# Large-scale transcriptome sequencing reveals novel expression patterns for key sex-related genes in a sex-changing fish

**DOI:** 10.1186/s13293-015-0044-8

**Published:** 2015-11-25

**Authors:** Hui Liu, Melissa S. Lamm, Kim Rutherford, Michael A. Black, John R. Godwin, Neil J. Gemmell

**Affiliations:** Department of Anatomy, University of Otago, Dunedin, New Zealand; Department of Biological Sciences, North Carolina State University, Raleigh, NC USA; W.M. Keck Center for Behavioral Biology, North Carolina State University, Raleigh, NC USA; Department of Biochemistry, University of Otago, Dunedin, New Zealand

**Keywords:** Sex-biased gene expression, Sexual dimorphism, Brain, Gonad, Transcriptome, RNA-seq, Protogynous sex change, Bluehead wrasse

## Abstract

**Background:**

Teleost fishes exhibit remarkably diverse and plastic sexual developmental patterns. One of the most astonishing is the rapid socially controlled female-to-male (protogynous) sex change observed in bluehead wrasses (*Thalassoma bifasciatum*). Such functional sex change is widespread in marine fishes, including species of commercial importance, yet its underlying molecular basis remains poorly explored.

**Methods:**

RNA sequencing was performed to characterize the transcriptomic profiles and identify genes exhibiting sex-biased expression in the brain (forebrain and midbrain) and gonads of bluehead wrasses. Functional annotation and enrichment analysis were carried out for the sex-biased genes in the gonad to detect global differences in gene products and genetic pathways between males and females.

**Results:**

Here we report the first transcriptomic analysis for a protogynous fish. Expression comparison between males and females reveals a large set of genes with sex-biased expression in the gonad, but relatively few such sex-biased genes in the brain. Functional annotation and enrichment analysis suggested that ovaries are mainly enriched for metabolic processes and testes for signal transduction, particularly receptors of neurotransmitters and steroid hormones. When compared to other species, many genes previously implicated in male sex determination and differentiation pathways showed conservation in their gonadal expression patterns in bluehead wrasses. However, some critical female-pathway genes (e.g., *rspo1* and *wnt4b*) exhibited unanticipated expression patterns. In the brain, gene expression patterns suggest that local neurosteroid production and signaling likely contribute to the sex differences observed.

**Conclusions:**

Expression patterns of key sex-related genes suggest that sex-changing fish predominantly use an evolutionarily conserved genetic toolkit, but that subtle variability in the standard sex-determination regulatory network likely contributes to sexual plasticity in these fish. This study not only provides the first molecular data on a system ideally suited to explore the molecular basis of sexual plasticity and tissue re-engineering, but also sheds some light on the evolution of diverse sex determination and differentiation systems.

**Electronic supplementary material:**

The online version of this article (doi:10.1186/s13293-015-0044-8) contains supplementary material, which is available to authorized users.

## Background

Sexual dimorphism is ubiquitous in nature: males and females differ not only in their gonadal structure and function, but also in many aspects of their morphology, physiology, and behavior [1–3]. While sex-determination mechanisms are relatively conserved in mammals and birds, teleost fishes show remarkably diverse sexual developmental patterns, including both genetic and environmental sex-determination (GSD and ESD) systems [1, 4, 5]. Such diversity probably arises from the extreme sexual plasticity characteristic of teleost fishes. For example, in fishes with GSD systems, sex is determined during early development stages and individuals remain in the same sex for a lifetime (defined as gonochorism) [4]. However, this primary sex differentiation guided by genetic signals can be interrupted or even reversed by temperature or endocrine-disrupting chemicals [6–11]. More extreme cases are found in fishes with ESD systems including sequential hermaphroditism in which some adults in a social group undergo functional sex change in response to environmental stimuli (e.g., temperature or social cues) [12–16]. Revealing the mechanisms underlying such sexual plasticity may help us understand how sex is maintained and gain insights into the origin and evolution of sex-determination systems.

The genetic bases of sexual dimorphism have been intensively studied for decades in mammals and birds, but are less well characterized in teleost fishes [1–4, 17, 18]. So far, genetic studies on sex determination in fishes have examined either sex-specific genetic differences or sex-biased gene expression [19–23]. The search for sex-specific genetic markers has not met with much success because, unlike mammals and birds, fishes have relatively young sex chromosomes that are not usually heteromorphic [24–28] with exceptions including some species of salmonids [29, 30], stickleback fishes [31], glass knife fishes [32], and half-smooth tongue sole, *Cynoglossus semilaevis* [33]. Even in fishes with heterogenic sex-determination systems, sex differences are usually limited to a few loci or certain linkage groups [34–39]. No conserved sex-specific gene has been found in teleost fishes: six sex-determining genes have been reported to evolve separately in different fish lineages [22, 40]. In contrast, studies examining sex-biased gene expression in fishes have yielded many more genes, including some that play conserved roles in vertebrate sex differentiation. However, for most of these sex-biased genes, their detailed molecular functions in fishes remain to be clarified [23, 41–46]. Studies to date have focused mainly on the expression patterns of a limited number of genes during primary sex differentiation stages in gonochoristic fishes [47–53]. Few studies have yet examined sex-biased gene expression in hermaphroditic fishes, with the exception of genetic studies in protandrous black porgy, *Acanthopagrus schlegelii* [19, 54], and transcriptomic studies in two other protandrous species: sharpsnout seabream, *Diplodus puntazzo*, and Asian seabass, *Lates calcarifer* [20, 21]. However, hermaphroditism is phylogenetically widespread in fishes, with protogyny being the most commonly observed pattern [15, 55, 56], but a large-scale analysis of sex-biased gene expression is currently lacking for protogynous fishes.

The bluehead wrasse, *Thalassoma bifasciatum*, is a diandric (two male phenotypes) protogynous species belonging to the wrasse family (Labridae) and is abundant on coral reefs throughout the Caribbean [57, 58]. This highly social species exhibits two major color phases: females and smaller sneaker males in initial phase (IP) share the same color pattern, while the large males display a distinct terminal phase (TP) phenotype [57–59]. Natural social groups typically consist of one dominant TP male and numerous females as well as a few IP males. Following the loss of the dominant TP male from a social group, both large females and IP males can transform into TP males through sex or role change, although the latter is rarely reported [55, 59, 60]. In females, functional gonadal sex change takes about a week while behavioral sex change can begin within minutes to hours [60, 61]. Importantly, manipulation of the social environment can induce sex change in females, which makes the bluehead wrasse a useful model for investigating sexual plasticity.

Significant progress has been made in understanding the ecology and the neuroendocrine bases of sex change in this species, but detailed mechanisms still remain elusive, especially at the molecular level [55, 62]. According to the Animal Genome Size Database [63], the haploid DNA contents (C-value) of bluehead wrasse is 0.98 picogram (1 picogram = 978 megabase pair). However, its genome and transcriptome sequences are not available yet. In this study, we took advantage of RNA sequencing technology and captured the transcriptomic profiles in the brain and gonads of TP male, female, and intersex bluehead wrasses. To identify genes exhibiting sex-biased expression in the brain (forebrain and midbrain) and gonads of the bluehead wrasse, we generated a *de novo* transcriptome assembly for read mapping and compared gene expression patterns at the isoform level between control females and TP males. We also conducted functional annotation and enrichment analysis on the genes showing sex-biased expression in the gonad to detect sex-biased genetic pathways that could contribute to gonadal sex differences in bluehead wrasses.

## Methods

### Sample collection

Sex change was induced in large females by the removal of dominant TP males from established social groups in the wild [61, 64, 65]. Twenty fishes were captured before or during the daily spawning period around high tide from patch reefs off the coast of Key Largo in late May 2012. All fishes were euthanized with an overdose of MS-222 (Sigma) within 2 min of capture, and the brain and gonads were dissected immediately. These experiments were performed in accordance with guidelines established by the Institutional Animal Care and Use Committee at North Carolina State University (NCSU).

One gonadal lobe and the whole brain were preserved in RNAlater (Life Technologies, Inc.) on ice, followed by storage at −20 °C for less than 1 week and transfer to −80 °C until RNA extraction. The other gonadal lobe was fixed in 4 % paraformaldehyde/1X PBS overnight at 4 °C, followed by storage in 1X PBS before being fixed in paraffin for histological sectioning and HE (hematoxylin and eosin) staining (Histology Laboratory, College of Veterinary Medicine, NCSU) to determine the gonadal status [66]. Before RNA extraction, the hindbrain (corpus cerebelli, pons, and medulla) was removed from each brain. Only the forebrain/midbrain was used for RNA sequencing, because the forebrain and midbrain contain regions belonging to the social behavior network and mesolimbic reward system, two neural circuits that are involved in the regulation of social decision-making [67], and thus may be key integrators and drivers of socially induced sex change.

### RNA extraction

The tissues were homogenized using TissueLyser II (QIAGEN®) (Center for Neuroendocrinology, Department of Anatomy, University of Otago). Forebrain/midbrain and gonadal total RNA were extracted with TRI reagent (Invitrogen) using chloroform (forebrain/midbrain) or bromochloropropane (gonads) as the phase separation reagent. Samples were then DNase-treated (TURBO DNA-free Kit, Ambion) and total RNA-cleaned (NucleoSpin RNA XS columns, Macherey-Nagel). RNA integrity was assessed on an Agilent 2100 Bioanalyzer. Sex-changing gonads consistently showed RNA profiles with a strong peak of low molecular weight RNA, which possibly corresponds to massive 5S RNA expression in atretic ovaries and masks the 18S and 28S rRNA peaks used for calculating RNA integrity numbers (RIN). Such patterns were also observed in ovaries and intersex gonads of thicklip gray mullets, *Chelon labrosus* [68], and ovaries of protandrous sharpsnout seabream, *Diplodus puntazzo* [20]. Therefore, RIN values could not serve as useful measures of RNA integrity in these sex-changing gonads of bluehead wrasses. For brain RNA, samples with RIN values above 6.0 were used for RNA-seq. Total RNA concentration was measured by Qubit 2.0 Fluorometer (Qubit RNA HS Assay Kit, Life Technologies), and samples were diluted to 10 ng/μL.

### RNA sequencing

Total RNA from 12 forebrain/midbrain and 12 gonadal samples (3 control females, 3 TP males, and 6 intersex fish), 500 ng per sample, were sent to the Otago Genomics and Bioinformatics Facility at the University of Otago under contract to New Zealand Genomics Limited for library construction and RNA sequencing. Twenty-four multiplexed libraries were prepared with the Illumina TruSeq Stranded mRNA Sample Prep Kit and 100-bp paired-end reads were generated using 8 flow cell lanes on the HiSeq 2000 platform. The insert size was designed to produce a small overlap between paired reads.

### Read pre-processing

Read quality was first assessed with FastQC (v0.10.1) [69]. Quality filtering was performed using Trimmomatic (v0.25) [70]: low quality reads were trimmed if average Phred quality scores were less than 20 within a 3-bp sliding window and discarded if the length was below 40 bp after trimming (Trimmomatic parameters: SLIDINGWINDOW:3:20 MINILEN:40). Read pairs were processed with FLASH (v1.2.4) [71]. Overlapping read pairs were joined and used for assembly along with the non-merged read pairs.

### *De novo* transcriptome assembly

Filtered short reads with high-quality scores were assembled *de novo* with Trinity [72] (r2014-03-23, default kmer 25, minimum contig length of 200 bp), an assembler developed for efficient and robust *de novo* reconstruction of transcriptomes from RNA-seq data [20, 73, 74]. Since our libraries were made using the dUTP method [75], we specified the library type by setting the “strand-specific library type (—SS_lib_type)” as “RF.” We also used the “—jaccard_clip” option to reduce chimeric fusion of transcripts [76].

#### Quality checking

Assembled contigs were first searched against CEGMA (Core Eukaryotic Genes Mapping Approach, v2.5) KOGs (the eukaryotic orthologous groups) [77]. We then ran “TransDecoder” (v1.0) [76] to check the chimeric rate in our assembly (if two large open reading frames were found in one contig, it would be reported as chimeric). Full-length transcript analysis was carried out using the Trinity function “analyze_blastPlus_topHit_coverage.pl” with BLAST+ (BLASTN, E-value cut-off 10^−50^) against 17 bluehead wrasse expressed sequence tags (ESTs) and 19,712 Nile tilapia protein sequences (Ensembl release 75) [76, 78, 79]. Finally, we manually checked the sequences of all the candidate genes based on read mapping and visualization in Integrative Genomics Viewer (IGV, v2.3.40) [80].

#### Annotation

The assembly was searched against the UniProt (Swiss-Prot and TrEMBL) protein database [81] with BLAST+ (BLASTX, E-value cut-off 10^−10^, keeping the top hit) [78] for taxonomic distribution and bacterial contamination detection. Information on taxa was obtained using an in-house Perl script, and the numbers of each taxon were manually checked.

We then conducted BLASTX searches of the assembled contigs against the Ensembl (release 75) Nile tilapia (*Oreochromis niloticus*), zebrafish (*Danio rerio*), and medaka (*Oryzias latipes*) protein databases (E-value cut-off 10^−10^, keeping the top hit) [78, 79].

Contigs with no hit in the protein databases were searched against the Ensembl (release 77) zebrafish non-coding RNA (ncRNA) database (BLASTN, E-value cut-off 10^−5^, keeping the top hit) and mapped to the tilapia genome downloaded from Ensembl (release 79, BLASTN, E-value cut-off 10^−10^) [78, 79]. Finally, putative open reading frames (ORFs) were searched in both annotated and unannotated contigs using OrfPredictor (v2.3) [82].

### Read mapping and differential expression analysis

The *de novo* transcriptome assembly served as a reference for read mapping. Raw reads were aligned to the assembly with Bowtie (v0.12.9) [83] and transcript abundance estimation was calculated with RNA-seq by expectation maximization (RSEM, v1.2.12) [84] using the align_and_estimate_abundance.pl script from the Trinity package [76]. RSEM expected counts for each contig (representing the isoform) were used for downstream differential expression analysis in R (v3.1.0) [85] using the DESeq package (v1.20.0) [86].

Comparisons between TP males and females were conducted separately for the brain and gonadal samples (3 samples for 2 conditions each) using the DESeq function nbinomTest [86]. Principal component analysis (PCA) [87] and the heatmap.2 function in the gplots package [88] were used to visualize global similarities and differences among either the brain or gonadal samples. Contigs with very low expression in either gonad or brain (average expected counts of mapped reads fewer than 1 per sample) were excluded prior to differential expression analysis to improve the statistical power [89]. All samples (including intersex samples) were used for estimating dispersions. *p* value adjustment was performed using the false discovery rate controlling procedure [90]. Contigs with an adjusted *p* value less than 0.05 and a fold change larger than 2 were reported as significantly differentially expressed between sexes in the gonad, while contigs with an adjusted *p* value less than 0.05 were reported as significantly differentially expressed between sexes in the brain.

### Gene ontology and pathway analysis

Contigs showing significantly sex-biased expression in the gonad were searched against the Ensembl zebrafish protein database (BLASTX, E-value cut-off 10^−10^). Matched zebrafish protein IDs were converted to unique Ensembl zebrafish gene IDs via BioMart [91]. These gene IDs were imported into the Database for Annotation, Visualization, and Integrated Discovery (DAVID, v6.7) [92] for functional annotation and enrichment analysis, using the default zebrafish database in DAVID (v6.7) as the background. Gene ontology (GO) [93] and pathway analysis [94] was carried out only for the gonad because there were not enough differentially expressed contigs detected in the brain. GO terms of level one and Kyoto Encyclopedia of Genes and Genomes (KEGG) pathways with a fold enrichment above 1.2 and *p* value below 0.05 are shown in Figs. 4 and 5. GO terms and KEGG pathways with *p* values below 0.05 after adjustment using the Benjamini and Hochberg (BH) procedure are indicated by stars.

## Results and discussion

### *De novo* transcriptome assembly

The Illumina HiSeq 2000 sequencing produced more than two billion 100-bp paired-end reads (1,106,170,692 read pairs). The raw sequence data in FASTQ format have been submitted to the National Centre for Biotechnology Information (NCBI) Sequence Read Archive (SRA) database and are accessible under accession number SRP06302. After trimming, 1,586,678,582 (71.7 %) high-quality reads were retained for the transcriptome assembly.

The *de novo* assembled transcriptome using Trinity [72] resulted in 230,626 contigs with a N50 of 1,146 bp and minimum and maximum contig lengths of 201 and 27,427 bp, respectively. There are 77,632 contigs having a length of 500 bp or more. Short contigs (<500 bp) were retained for annotation and mapping because many neuropeptides have a short protein sequence.

Assembly quality was assessed by three means: the representation of core eukaryotic genes, predicted chimeric rate, and full-length recovery of the bluehead wrasse expressed sequence tags (ESTs) and Nile tilapia protein sequences (Ensembl release 75) [79]. All CEGMA KOGs (the eukaryotic orthologous groups) [77] were present in this assembly (98 % complete, 100 % partial). The predicted chimeric rate [76] was 3.2 %. All of the bluehead ESTs were recovered (BLASTN, E-value ≤10^−50^): 14 ESTs with >90 % recovery and 3 ESTs with 70–80 % recovery. Fifty-eight percent of Nile tilapia protein sequences had a match in the bluehead wrasse transcriptome assembly with alignment coverage above 90 % (BLASTX, E-value ≤10^−10^).

### Transcriptome annotation

**Fig. 1:**
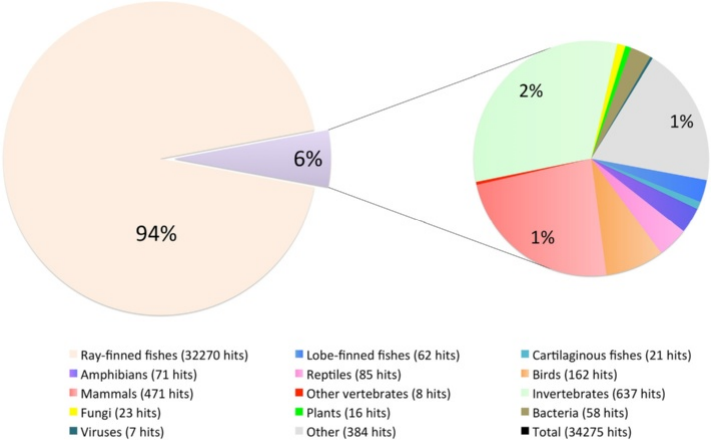
Taxonomic distribution of top BLASTX hits in UniProt protein database. Numbers of unique hits of protein sequences in each group are shown in the *parentheses*

The bluehead wrasse transcriptome assembly was searched against the UniProt (Swiss-Prot and TrEMBL) protein database [81] (BLASTX, E-value ≤10^−10^, keeping the top hit). In total, 41,799 contigs (18 %) had significant hits to 34,275 unique protein sequences. Of these sequences, 94 % came from ray-finned bony fishes while only 63 contigs matched to bacterial sequences (Fig. 1). This indicates negligible contamination of bacteria, which is consistent with expectations that our assembly comprises mainly brain and gonadal coding RNAs.

Searching against the Ensembl protein databases [79] (BLASTX, E-value ≤10^−10^, keeping the top hit), we found 16–17 % of the assembled contigs had a significant BLASTX match (E-value ≤10^−10^, keeping the top hit). Of 26,763 input Nile tilapia protein sequences, 20,182 sequences were found in our assembly (Table 1). Similar results have been reported in two other non-model fish transcriptomes [20, 95].

**Table 1:** Annotation of the *de novo* transcriptome assembly

Species	Nile tilapia	Zebrafish	Medaka	Zebrafish
Ensembl database type	Protein	Protein	Protein	ncRNA
Contigs with hits	38,606	37,149	36,695	168
Unique hits	20,182	20,496	17,994	149
Input sequences	26,763	43,153	24,674	8319
Cut-off E-value	10^−10^	10^−10^	10^−10^	10^−5^

**Fig. 2:**
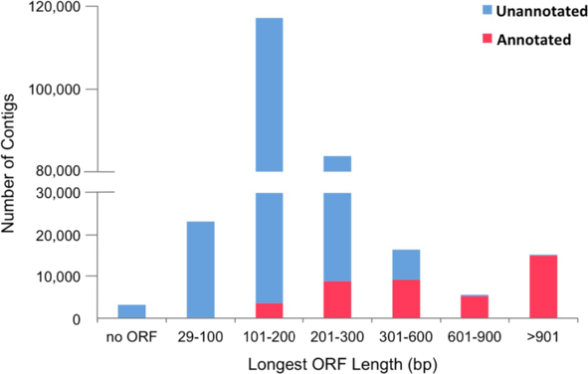
Length distribution of the longest ORFs in assembled contigs (*blue*: unannotated contigs, *pink*: annotated contigs)

Surprisingly, a large portion (82 %) of the contigs had no significant BLASTX match to any known protein sequence. These sequences were searched against the Ensembl zebrafish ncRNA database [79] (BLASTN, E-value ≤10^−5^, 8819 input ncRNA sequences), but only 93 contigs had a match, including processed or antisense transcripts with no protein product, miRNA, miscRNA, snoRNA etc. Putative ORFs were searched in both annotated and unannotated contigs using OrfPredictor [82]. The length distribution of the longest ORFs is shown in Fig. 2. Briefly, over 98 % of the contigs contained ORFs, but most (69 %) were smaller than 300 bp. Almost all of the unannotated contigs (>99 %) had an ORF smaller than 600 bp. These contigs may represent novel protein-coding transcripts, fragmented UTRs, pre-mature mRNA sequences with retained introns, or polyadenylated non-coding RNAs of potential biological importance. At present, however, it is still challenging to provide complete annotations for a *de novo* assembled transcriptome, especially for a non-model teleost fish for which few genomic resources are available. Multiple BLAST searches provide a powerful means for automated annotations but are limited by available sequences, sequence similarity among homologues, and alignment sensitivity. Future genome sequencing and more information on alternatively spliced isoforms and non-coding RNAs will improve our current annotation. It will be useful to revisit these data as more gene sequences become available.

### Differential gene expression between female and TP male

All of the contigs were kept for read mapping, and the RSEM [84] expected value table of contigs (representing isoforms) was used for expression analysis. This allows detection of isoform-specific expression patterns and clustering of the contigs based on both annotations and expression patterns.

In total, 889,652,430 raw read pairs (from control females and TP males) were mapped back to the reference transcriptome assembly [83]. In this paper, we focused on the sex-biased gene expression; thus, we only report the comparison between females and TP males.

#### Global gene expression patterns in the brain and gonad

**Fig. 3:**
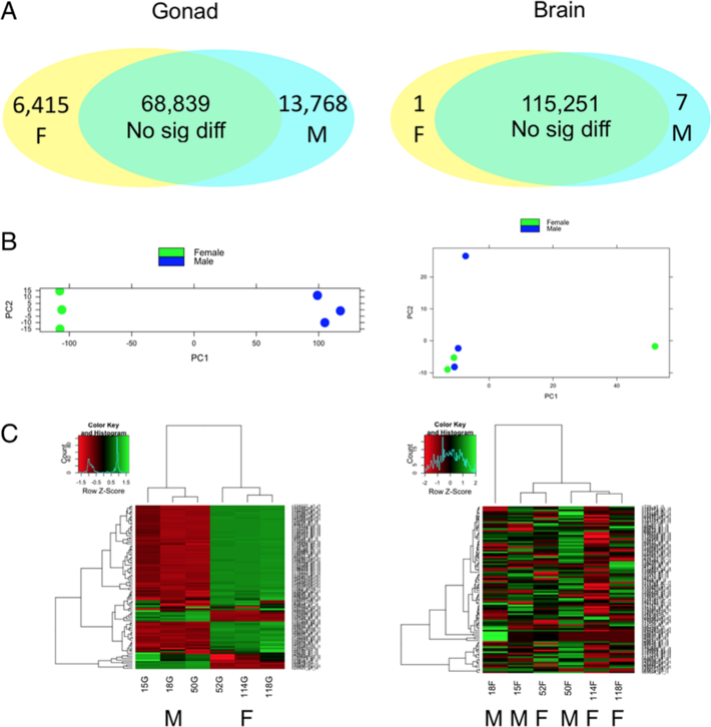
Gene expression patterns in the brain (*right*) and gonads (*left*). **a** Numbers of differentially expressed contigs between TP males (*M*) and females (*F*). **b** PCA plots of brain and gonadal samples (*green*: female, *blue*: TP male). **c** Heatmaps showing the expression of top 100 contigs in brain and gonads (ordered by average normalized read counts across the row; *red*: lower expression, *green*: higher expression; *M*: TP male, *F*: female)

Comparisons between TP males and females were conducted separately for the brain and gonadal samples (3 samples for 2 conditions each) using the DESeq function nbinomTest [86]. *p* value adjustment was performed using Benjamini and Hochberg (BH) procedure [90]. Principal component analysis (PCA) plots [87] and heatmaps [88] both showed that sex differences in gene expression were much more pronounced between the ovary and testis than those between male and female brains (Fig. 3b, c). Consistently, a large number of contigs showed sex-biased expression in the gonad (fold change ≥2 and BH adjusted *p* value ≤0.05), while only eight contigs showed sex-biased expression in the brain (BH adjusted *p* value ≤0.05). Similar global expression patterns were also reported in zebrafish [44, 96] and sharpsnout seabream [20], which may reflect the functional and regulatory differences between the brain and gonads.

#### A large set of genes showed significant sex-biased expression in the gonad

Expression analysis revealed a large set of transcripts differentially expressed between ovary and testis of the bluehead wrasse (fold change ≥2 and BH adjusted *p* value ≤0.05). Contigs showing male-biased expression were twice as abundant as those showing female-biased expression, although contigs with the highest expression in the gonad were female-biased (Fig. 3a, c). Of the 13,768 male-biased contigs, 6769 (49 %) contigs had a significant BLASTX match in the UniProt protein databases (E-value ≤10^−10^), including 6279 hits in the Ensembl zebrafish protein database. Of the 6415 female-biased contigs, 4246 (66 %) had a significant BLASTX match in the UniProt protein databases (E-value ≤10^−10^), including 4074 hits in the Ensembl zebrafish protein database.

#### Enriched gene ontology terms and pathways in testis and ovary

Contigs showing sex-biased expression in the gonad were mapped to the Ensembl zebrafish protein database and further converted to their equivalent Ensembl zebrafish gene IDs (4824 male-biased, 3373 female-biased) via BioMart [91]. These gene IDs (Additional file 1) were searched against the DAVID (v6.7) [92] zebrafish database to detect which GO terms [93] and KEGG pathways [94] were enriched in the testis and ovary of bluehead wrasses, respectively. As a result, 4080 (male-biased) and 2989 (female-biased) DAVID IDs were reported, of which 30–50 % were assigned with GO terms and about 20 % were mapped to KEGG pathways.

**Fig. 4:**
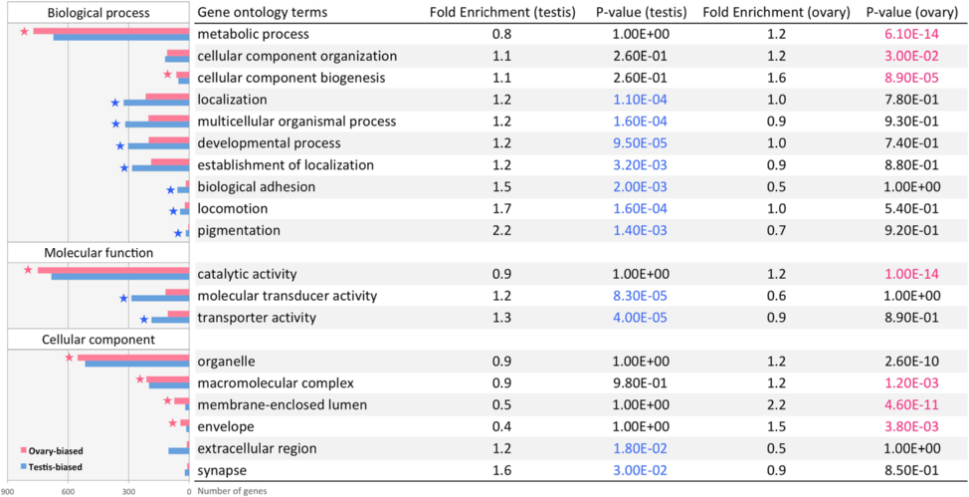
Top GO terms enriched in the ovary (*pink*) and testis (*blue*). Enriched GO terms with modified Fisher exact test *p* value below 0.05 and fold enrichment above 1.2 are shown here. *Stars* indicate GO terms with BH adjusted *p* value below 0.05

**Fig. 5:**
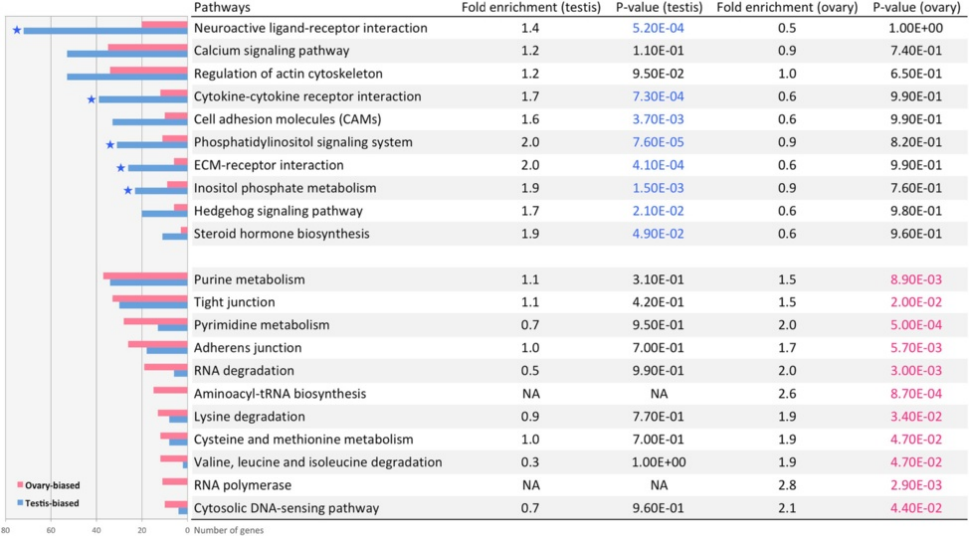
KEGG pathways enriched in the ovary (*pink*) and testis (*blue*). Enriched pathways with modified Fisher exact test *p* value below 0.05 and fold enrichment above 1.2 are shown here. *Stars* indicate pathways with BH adjusted *p* value below 0.05

Significantly enriched GO terms (level 1) in the ovary and testis are shown in Fig. 4. In general, the ovary was enriched for metabolic process, while the testis was enriched for signal transduction and receptor activity. Similarly, the pathway enrichment analysis also found that ovaries are enriched for RNA and protein metabolism, while testes are enriched for signal transduction (Fig. 5).

**Fig. 6:**
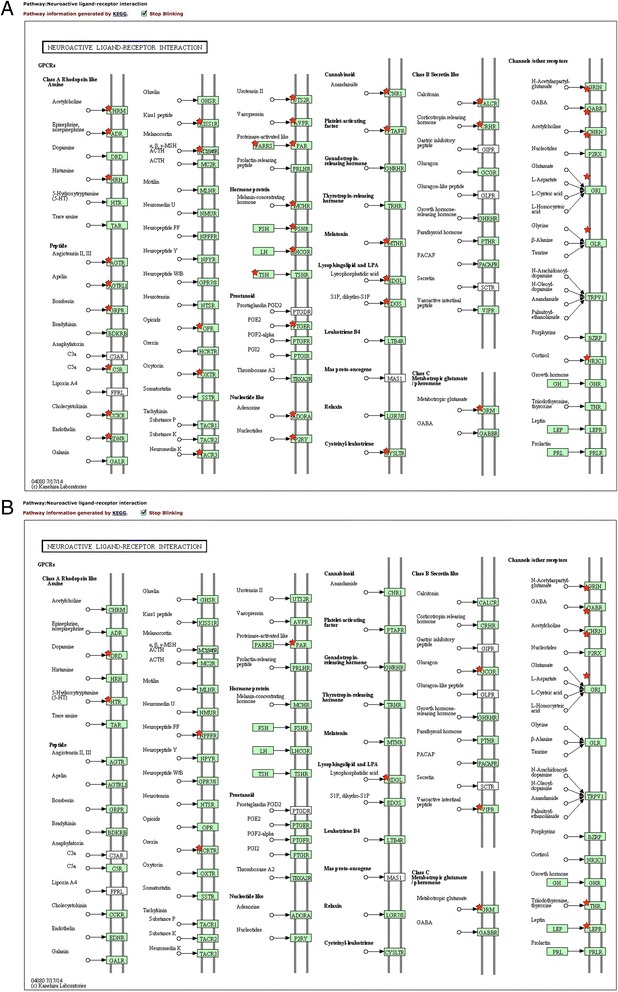
The neuroactive ligand-receptor interaction pathway was significantly enriched in the testis (**a**) but not in the ovary (**b**). *Red stars* indicate the DE genes significantly up-regulated (fold change above 2 and BH adjusted *p* value below 0.05) in the testis (**a**) or the ovary (**b**)

Interestingly, the top pathway enriched in the testis was “neuroactive ligand-receptor interaction” (Fig. 4), which includes receptors for many neuropeptides (Fig. 6a, b). Within this pathway, receptors of norepinephrine, epinephrine, melatonin, oxytocin/isotocin (IT), and vasopression/vasotocin (AVT) were significantly over-expressed in the testis (Fig. 6a) while receptors of dopamine, serotonin (5-HT), and neuropeptide FF were significantly over-expressed in the ovary (Fig. 6b). Some of these neuropeptides have been suggested to play important roles at the onset of protogynous sex change [55, 56, 62]. Briefly, norepinephrine and vasotocin have a promoting effect on gonadal or behavioral sex change, while dopamine and serotonin have an inhibitory effect [97–100]. The function of isotocin and melatonin in sex-changing fishes are largely unknown due to limited information. Nevertheless, the interesting point here is that these neuropeptides were thought to act on the brain and regulate gonadal sex change indirectly through the hypothalamic-pituitary-gonadal (HPG) axis [55, 101, 102]. However, their receptors are widely expressed in the gonad. Some recent studies suggest these neuropeptides can act directly on the gonad, in addition to their classical actions through the HPG axis. For example, vasotocin and melatonin are reported to regulate oocyte maturation in catfish [103] and carp [104], whereas vasotocin and catecholamines (dopamine, norepinephrine, and epinephrine) are shown to modulate ovarian steroidogenesis in catfish in a biphasic manner [103, 105]. Further studies on expression and function of these neuropeptides in both the brain and gonads of sex-changing fishes are warranted.

**Fig. 7:**
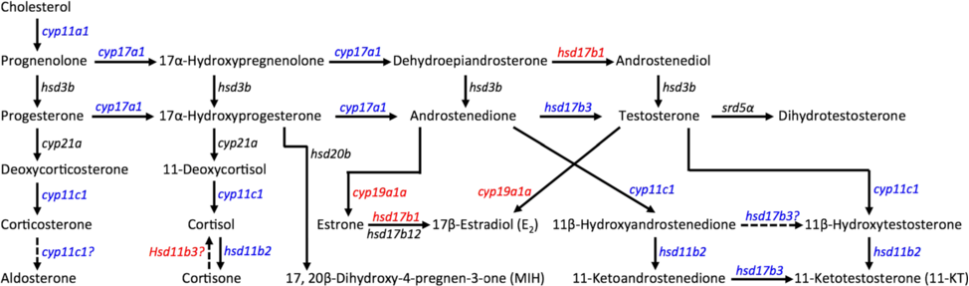
Postulated pathways of steroidogenesis in the gonad (adapted from [118, 178, 179]). Putative catabolic activities are indicated by *arrows* with *dash lines*. Genes showing male- or female-biased expression in bluehead wrasse gonads are colored in *blue* or *red*, respectively

**Fig. 8:**
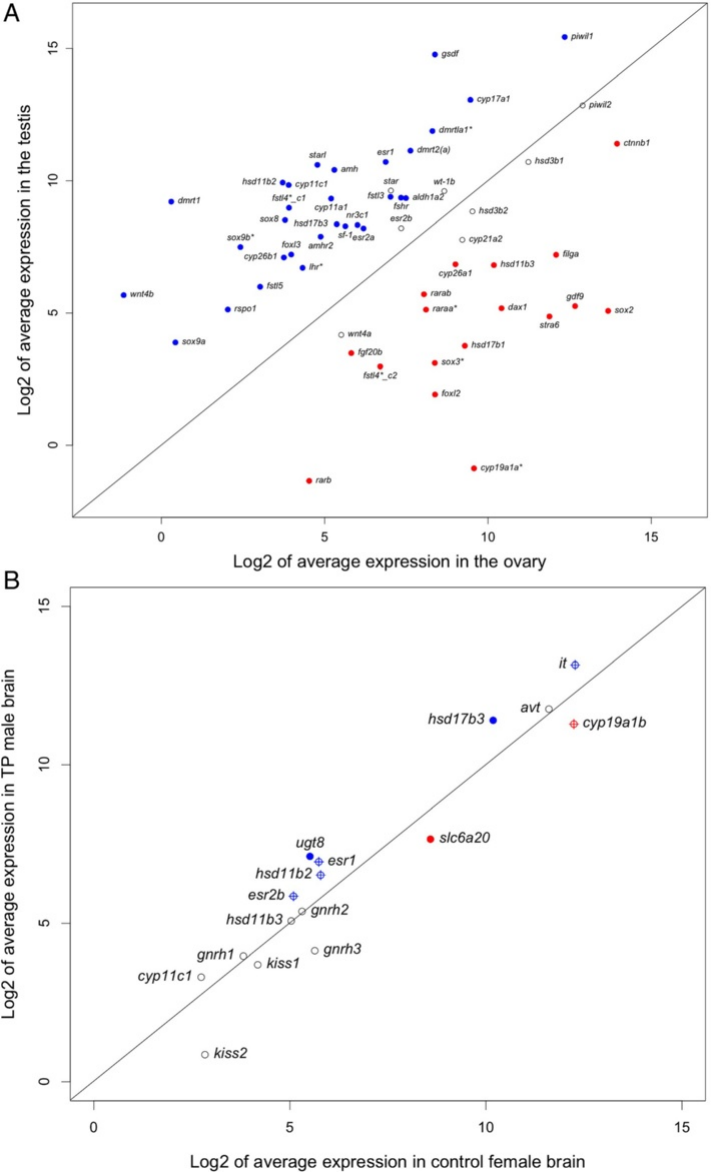
Expression patterns of candidate genes in the gonad (**a**) and brain (**b**). **a** Expression patterns of 56 sex-related genes in the gonad of bluehead wrasses. Genes with BH adjusted *p* value below 0.05 are shown in *solid circles* (*blue*: male-biased, *red*: female-biased). **b** Expression patterns of 16 genes of interest in TP male and female forebrain/midbrain of bluehead wrasses. Genes (*hsd17b3*, *ugt8*, *slc6a20*) with BH adjusted *p* value below 0.05 are shown in *solid circles*. Genes (*it*, *cyp19a1b*, *esr1*, *esr2b*, and *hsd11b2*) with pre-adjusted *p* values below 0.05 prior to BH correction are shown in *open circles* with a *cross*. Genes (*avt*, *hsd11b3*, *cyp11c1*, *gnrh1*, *gnrh2*, *gnrh3*, *kiss1*, and *kiss2*) with *p* values above 0.05 before and after BH correction are shown in *open circles*. Genes showing male- or female-biased expression are colored in *blue* or *red*, respectively

Another pathway enriched in the testis is related to steroid hormone biosynthesis (Fig. 4). Steroid hormones are known to play a critical role in sex differentiation across vertebrates [4]. Within this pathway, only three genes (*cyp19a1a*, *hsd11b3*, *hsd17b1*) showed significantly female-biased expression in the gonad while 11 genes showed significantly male-biased expression, including *cyp11c1*, *hsd11b2*, *hsd17b3*, *cyp11a1*, and *cyp17a1* (Figs. 7 and 8a). These results are generally consistent with our current knowledge of sexually dimorphic levels of steroid hormones in fish.

In teleost fishes, 17β-estradiol (E_2_) and 11-ketotestosterone (11-KT) function as the major estrogen and androgen, respectively [4]. Testosterone (T) serum levels can also be high in males and females, but T can be converted into either E_2_ by aromatase (*cyp19a1a* in the gonad) or 11-KT by 11β-hydroxylase (*cyp11b* or *cyp11c1* in zebrafish) and 11β-hydroxysteroid dehydrogenase 2 (*hsd11b2*) [4, 106, 107]. In protogynous species, steroid hormones play a central role in controlling sex change: high plasma E_2_ levels prevent females from changing into males, whereas blocking E_2_ production or injecting 11-KT in females can induce sex change [108]. As expected in our study, *cyp19a1a* expression was only detected in the ovary, whereas *cyp11c1* and *hsd11b2* expression were significantly higher in the testis (Fig. 8a, Table 2, and Additional file 2). The brain aromatase gene *cyp19a1b* was also detected in the gonad of rainbow trout [109], but our data showed almost no expression of *cyp19a1b* in bluehead wrasse gonads. In addition, the genes that encode androgen receptors (*ar1* and *ar2*) showed no sex-biased expression, while the estrogen receptor genes (*esr1*, *esr2a*, and *esr2b*) had higher expression in the testis, although the male-biased expression of *esr2b* was not statistically significant (Fig. 8a, Table 2, and Additional file 2). Such male-biased expression of estrogen receptors has been shown during late sexual differentiation for Nile tilapia (70dah) [49] and rainbow trout (60-110dpf) [110]. A recent study on rainbow trout also showed significantly elevated testicular expression of *esr1a* and *esr2a* during the final stage of spermiation, while *esr1b* and *esr2b* were expressed at early stages of testicular development [111]. In the same study, androgen implants up-regulated testicular *esr1a*, *esr2a*, and *esr2b* expression but down-regulated *cyp19a1a* expression, whereas estrogens reduced testicular *cyp19a1a* expression but increased the expression of *cyp19a1b* and *esr1b*. These findings all suggest a potential role for estrogens and their receptors in teleost testicular development.

**Table 2:** Genes showing sex-biased expression in the gonad

Gene symbol	Gene description	Contig ID	Expression in bluehead wrasse	Expression in other fishes
Steroidogenesis and hormone receptors				
*cyp19a1a* ^a^	Aromatase a (gonad isoform)	c75632_g1_i1	F	F [19, 20, 47, 110, 137, 180, 181]
*cyp11c1/b2*	Steroid 11β-hydroxylase	c112162_g1_i1	M	M [20, 21, 49, 137]
*hsd11b2*	11β-Hydroxysteroid dehydrogenase type 2	c117833_g1_i1	M	M [21, 47]
*hsd11b3*	11β-Hydroxysteroid dehydrogenase type 3 (or 1 like a)	c71474_g1_i1	F	NSD [47]
*cyp17a1*	Steroid 17α-hydroxylase 1	c152588_g2_i1	M	M [21, 47, 110]
*hsd17b1*	17β-Hydroxysteroid dehydrogenase type 1	c33723_g1_i1	F	F [47]
*hsd17b3*	17β-Hydroxysteroid dehydrogenase type 3	c3610_g1_i1	M	NSD [47]
*cyp11a1*	Cholesterol side-chain cleaving enzyme	c152363_g1_i1	M	M [47]
*star-like*	Steroidogenic acute regulatory protein	c156452_g1_i1	M	M [47]
*esr1*	Estrogen receptor alpha	c73327_g1_i1	M	M [21, 47, 110]
*esr2a*	Estrogen receptor beta 1	c70616_g1_i1	M	F [47]
*esr2b*	Estrogen receptor beta 2	c110359_g1_i1	M but NSD	M [47, 182]
*fshr*	Follicle-stimulating hormone receptor	c152458_g1_i1	M	M [183] or NSD [182]
*lhr* ^a^	Luteinizing hormone receptor	c74538_g1_i1	M	NSD [182]
*nr3c1*	Glucocorticoid receptor	c4332_g1_i1	M	
*nr3c2*	Mineralocorticoid receptor	c1060_g1_i1	F	
Key sex-related transcription factors				
*dmrt1*	Doublesex- and mab-3-related transcription factor 1	c154918_g1_i1_split_1	M	M [21, 49, 137, 184–188]
*dmrt2(a)*	Doublesex- and mab-3-related transcription factor 2(a)	c155062_g1_i1	M	
*dmrt1a1* ^a^	Doublesex- and mab-3-related transcription factor like A1	c90860_g1_i1	M	
*foxl2*	Forkhead box L2	c29733_g1_i1	F	F [19, 46, 49, 110, 125, 127, 133] or NSD [21, 135]
*foxl3*	Forkhead box L3	c158785_g1_i1	M	M [125]
*sf-1*	Steroidogenic factor-1	c152032_g1_i1	M	M [20, 49, 110]
*amh*	Anti-Müllerian hormone or Müllerian-inhibiting substance	c115827_g1_i1	M	M [20, 21, 49, 189]
*amhr2*	Anti-Müllerian hormone receptor 2	c197093_g1_i1	M	M [34, 189]
*gsdf*	Gonadal soma derived factor	c69648_g1_i1-a	M	M [48, 190]
*sox2*	SRY-related HMG box 2	c32495_g1_i1	F	
*sox3* ^a^	SRY-related HMG box 3	c72647_g1_i1	F	F [191] or M [186, 192]
*sox8*	SRY-related HMG box 8	c75695_g1_i1	M	M [193]
*sox9a*	SRY-related HMG box 9a	c4248_g1_i1	M	M [49, 110, 185, 191, 194] or F [137, 195]
*sox9b* ^a^	SRY-related HMG box 9b	c193758_g2_i1	M	M [196] or F [137, 185, 194]
*wt-1a* ^a^	Wilms tumor protein 1a	c32767_g1_i1	M	M [20, 21] or F [137]
*wt-1b*	Wilms tumor protein 1b	c29263_g1_i1	M but NSD	M [20] or F [137]
*dax1/nr0b1*	Dosage-sensitive sex reversal, adrenal hypoplasia critical region, on chromosome X, gene 1	c209037_g1_i1	F	M [49] or F [137, 197]
*gdf9*	Growth and differentiation factor 9	c152091_g1_i1	F	F [110]
*fgf20b*	Fibroblast growth factor 20-like	c193577_g1_i1	F	F [47]
*figla*	Factor in the germline alpha	c204647_g1_i1	F	F [137, 184]
Rspo1/Wnt4/β-catenin pathway				
*wnt4a*	Wingless-type MMTV integration site family, member 4a	c167432_g1_i1	F but NSD	F [19, 137] or M [141]
*wnt4b*	Wingless-type MMTV integration site family, member 4b	c203717_g1_i1	M	NSD [141] or M [137]
*rspo1*	R-spondin-1 (precursor)	c155259_g1_i1	M	F [142] or M [137]
*ctnnb1*	Catenin (cadherin-associated protein), beta 1	c70814_g1_i1	F	F [20, 21, 137]
*fstl3*	Follistatin-like 3	c85803_g1_i1	M	
*fstl4* ^a^ *_c1*	Follistatin-like 4_contig1	c6910_g1_i1	M	
*fstl4* ^a^ *_c2*	Follistatin-like 4_contig2	c76818_g1_i1	F	
*fstl5*	Follistatin-like 5	c110224_g1_i1	M	
Retinoid acid signaling pathway				
*aldh1a2*	Aldehyde dehydrogenase 1 family, member A2	c158408_g1_i1	M	M [145]
*cyp26a1*	Cytochrome P450, family 26, subfamily a, polypeptide 1	c29815_g1_i1	F	F [21, 145]
*cyp26b1*	Cytochrome P450, family 26, subfamily b, polypeptide 1	c117560_g1_i1	M	M [21] or NSD [145]
*raraa*	Retinoid acid receptor alpha a	c199432_g1_i1	F	
*rarab*	Retinoid acid receptor alpha b	c153705_g1_i1	F	
*rarb*	Retinoid acid receptor beta	c209577_g1_i1	F	
*stra6*	Stimulated by retinoic acid gene 6	c153800_g1_i1	F	F but NSD [21]
Epigenetic regulatory factors				
*piwi-like1*	P-element induced wimpy testis (piwi) like 1	c1516_g1_i1	M	M [21]
*dnmt1*	DNA methyltransferase 1	c30017_g1_i1	F	
*dnmt3aa*	DNA methyltransferase 3aa	c755_g1_i1	M	
*dnmt3ab* ^a^ *_c1*	DNA methyltransferase 3ab_contig 1	c193863_g1_i1	F	
*dnmt3ab* ^a^ *_c2*	DNA methyltransferase 3ab_contig 2	c157308_g1_i1	M	
*dnmt3b*	DNA methyltransferase 3b	c71358_g1_i1	M	
*dnmt3*	DNA methyltransferase 3	c194062_g1_i1	F	
*dnmt4*	DNA methyltransferase 4	c161106_g1_i1	M	
*hdac2* ^a^	Histone deacetylase 2	c75925_g1_i1	F	
*hdac7*	Histone deacetylase 7	c193969_g1_i1	F	
*hdac8*	Histone deacetylase 8	c71086_g1_i1	M	
*hdac10*	Histone deacetylase 10	c37022_g1_i1	F	
*hdac11* ^a^ *_c1*	Histone deacetylase 11_contig 1	c152723_g1_i1	M	
*hdac11* ^a^ *_c2*	Histone deacetylase 11_contig 2	c115912_g1_i1	F	
*Ep300a* ^a^	Histone acetyltransferase—E1A binding protein 300a	c193959_g1_i1	F	
*Ep300b* ^a^	Histone acetyltransferase—E1A binding protein 300b	c112702_g1_i1	F	
*KAT2b*	Histone acetyltransferase—K(lysine) acetyltransferase 2b	c152736_g1_i1	M	
*KAT7* ^a^	Histone acetyltransferase—K(lysine) acetyltransferase 7	c72324_g1_i1	M	

*M* male-biased, *F* female-biased, *NSD* not significantly different

^a^Indicates genes that have more than one contigs: only the longest contig of each gene is shown in this table

Interestingly, *cyp11c1* and *hsd11b2* are also involved in cortisol (or glucocorticoid, GC) production: Cyp11c1 converts 11-deoxycortisol to cortisol while Hsd11b2 converts cortisol to its inactive form cortisone, and Hsd11b3 (or Hsd11b1-like) could convert cortisone to cortisol [107, 112]. Cortisol treatment has been reported to cause masculinization of genetic females of medaka [113, 114], Japanese flounder [2, 115], southern flounder [116], and pejerrey [117]. In Japanese flounder, cortisol was suggested to cause female-to-male sex reversal by suppressing *cyp19a1a* expression [20, 115]. In zebrafish [118] and pejerrey [112], cortisol treatment of larvae elevated *hsd11b2* expression, while cortisol also enhanced *in vitro* 11-KT synthesis in pejerrey testes. Sexually dimorphic expression of *cyp11c1*, *hsd11b2*, *hsd11b3*, and *nr3c1* (nuclear receptor subfamily 3, group C, member 1 or glucocorticoid receptor) found in the gonad of bluehead wrasse (Fig. 8a, Table 2, and Additional file 2) suggests that local cortisol production could be important for gonadal sex differences. Moreover, cortisol treatment can induce protogynous sex change in three-spot wrasse [119], but a peak in serum cortisol levels appears to be a key event during gonadal sex change in both protandrous and protogynous species [120, 121]. The specific role of cortisol in regulating gonadal sex change remains to be clarified.

#### Expression patterns of genes involved in sex determination/differentiation

The processes of sex determination and differentiation can be viewed as a battle for primacy between a male regulatory gene network (e.g., *dmrt1*, *sf-1*, *amh*, *sox9*) and female genetic pathways involving *foxl2* and Rspo1/Wnt/β-catenin signaling [122, 123]. Despite the diverse regulatory mechanisms, expression patterns of these genes are generally consistent across taxa [1, 40, 122, 124]. In our study, male-pathway genes all showed significantly higher expression in the testis (e.g., *dmrt1*, *sf-1*, *amh*, *amhr2*, *sox9a*/*b*, *sox8*, and *gsdf*). In contrast, a few genes involved in the female-pathway (e.g., *rspo1*, *wnt4b*) showed unexpected expression patterns (Fig. 8a, Table 2, and Additional file 2).

First, two paralogues of forkhead box L2 genes (referred to as *foxl2* and *foxl3*) were detected in the gonad of the bluehead wrasse. *Foxl2* and *foxl3* probably originated from an ancient genome duplication event; they are present ubiquitously in fish lineages but *foxl3* was repeatedly lost in the tetrapods [125]. Foxl2 is critical for maintenance of female differentiation [126, 127] and can up-regulate *cyp19a1a* expression together with Sf-1 [128]. Foxl3 was proposed to play a role in testicular development, but its exact function remains elusive. In our study, *foxl2* was expressed at higher levels in the ovary while *foxl3* expression was higher in the testis of bluehead wrasses (Fig. 8a, Table 2, and Additional file 2). Such sexually dimorphic expression was also found in European sea bass, and the gonadal expression of *foxl2* and *foxl3* varied significantly during the reproductive cycles [125].

In recent years, Dmrt1 (doublesex and mab-3 related transcription factor 1) has received much attention due to its conserved role in vertebrate testicular differentiation and maintenance [127, 129–132]. *Dmrt1* expression was significantly higher in the testis than in the ovary of the bluehead wrasse (Fig. 8a, Table 2, and Additional file 2). Dmrt1 and Foxl2 have been proposed to have antagonistic effects on *cyp19a1a* expression to control gonadal sex fate [124, 127]. This hypothesis has been supported by studies in tilapia where knockout of *cyp19a1a* or *foxl2* expression caused gonadal sex reversal in females while *dmrt1* and *cyp11b2* (11β-hydroxylase) were co-expressed in follicular cells surrounding the degenerating oocytes [127]. Moreover, *foxl2* expression decreased while *dmrt1* expression increased during female-to-male sex change in honeycomb grouper [133]. However, such shifts in *foxl2* and *dmrt1* expression did not occur until the late transitioning stage, which was downstream of declining E_2_ levels [134]. *Foxl2* showed no strong sexually dimorphic expression in the gonad of protogynous three-spotted wrasses, and its expression even increased during aromatase-inhibitor-induced sex change [135]. Thus, the roles of *foxl2* and *dmrt1* may be species-specific in sex-changing fishes. Further manipulative studies will be especially useful for elucidating the precise functions of these key genes in sex-changing fishes.

Most genes involved in the ovary-specific Rspo1/Wnt/β-catenin signaling pathway showed sexually dimorphic expression in the gonad of bluehead wrasses (Fig. 8a, Table 2, and Additional file 2). However, some of these genes displayed an expression pattern that was opposite to our expectations based on studies from mammalian models [17, 136]. For example, *ctnnb1* (β-catenin) was highly expressed in both the ovary and testis of bluehead wrasse, but its expression was significantly female-biased. In contrast, *rspo1* (R-spondin-1) and *wnt4b* (wingless-type MMTV integration site family, member 4b) were expressed at much lower levels in the bluehead wrasse gonads, but they both showed significantly male-biased expression. Similar sexually dimorphic expression patterns of *ctnnb1*, *rspo1*, and *wnt4b* were also reported in east cichlid fishes [137]. Fst (follistatin) is downstream to Wnt4 signaling [17, 136, 138]. We detected a few *fst-like* genes in the bluehead wrasse gonad: *fstl3* and *fstl5* both showed male-biased expression, while two long isoforms of *fstl4* showed either female- or male-biased expression. It has been well-established in mammals that Rspo1, β-catenin, Wnt4, and Fst are key players in early ovarian differentiation [17, 136, 139], but information is limited regarding their roles in teleost fishes. Research to date, including our current study, reveals complicated expression patterns of these genes in fishes [18, 20, 53, 140–143]. Collectively, these data suggest *ctnnb1* likely plays a conserved role in both establishing and maintaining female sex differentiation across vertebrate taxa, while other genes involved in Rspo1/Wnt/β-catenin signaling pathway may participate in both ovarian and testicular development in fishes. More manipulative studies are needed to better characterize the roles of these genes in teleost fishes and to test whether the male-biased expression of *rspo1* and *wnt4b* is involved in protogynous sex change.

The RA (retinoid acid) signaling pathway is important in ovarian differentiation because RA controls the sex-specific timing of meiosis initiation [144–146]. RA level is regulated by Aldh1a (retinal dehydrogenase) and Cyp26 enzymes: Aldh1a2 increases RA level and initiates meiosis, while Cyp26a1 and Cyp26b1 decrease RA level and prevent germ cells from entering into meiosis [145]. Our study revealed higher expression of *aldh1a2* and *cyp26b1* but lower expression of *cyp26a1* and genes encoding RA receptors (*raraa*, *rarab*, and *rarb*) in the testis of bluehead wrasses (Fig. 8a, Table 2, and Additional file 2). These patterns are consistent with findings in Nile tilapia [145] and mice [147]. In addition, Cyp26b1 prevents *stra8* (stimulated by retinoic acid gene 8) expression in mouse testes [147, 148]. S*tra8* is lost in teleost fishes [149], but we found *stra6*, the receptor for retinol-binding protein 4 [150], in bluehead wrasse gonads. Its expression is much lower in the testis than in the ovary, which is consistent with high expression of *cyp26b1* in the testis (Fig. 8a, Table 2, and Additional file 2). Interestingly, studies in mice suggest that *dmrt1* expression is essential to maintain male-sex fate because it can protect the testis from transdifferentiation into ovary by RA signaling [131, 132]. Another study in mice also supports the hypothesis that Sox9 and Sf-1 up-regulate *cyp26b1* to maintain the male fate of germ cells in testes, while Foxl2 acts to antagonize *cyp26b1* expression in ovaries [151]. Taken together, the RA signaling pathway may play a key role in regulating gonadal sex change in hermaphroditic fishes and warrants further investigation.

Lastly, accumulating evidence suggests that epigenetic modifications also participate in the regulation of sex differentiation and sex change [152–157]. Transcripts of mRNA encoding DNA methyltransferases (Dnmt) and histone deacetylases (Hdac) or acetyltransferases (Hat) were detected in the bluehead wrasse, and most showed sex-biased expression in the gonad (Table 2, and Additional file 2). However, because the epigenetic mechanisms underlying sex differentiation are still poorly understood, we cannot infer any detailed functions of these genes from their expression patterns. Future studies are needed to reveal their molecular functions in sex differentiation and sex change.

#### Few sex-biased genes detected in the forebrain/midbrain

The brain represents a key site where environment stimuli and internal signals are integrated to regulate vertebrate physiology and behavior. Sex differences in the brain have been a major and growing focus in neuroscience [2, 3]. In mammals, sex differences in the brain are likely established by both organizational effects of sex steroid hormones and cellular autonomous regulation based on sex chromosomes [2]. Teleost brains, however, appear to show less sex bias in brain structure or gene expression [20, 45, 96]. Thus, the sex differences observed in teleost brains may be due primarily to the activational influences of steroid hormones [158], which may also explain the brain sexual lability of teleost fishes.

In our study, expression analysis using the DESeq package revealed seven up-regulated contigs and one down-regulated contig in the TP male bluehead wrasse forebrain/midbrain (Additional file 3). Only four of these contigs had a significant BLASTX match in the UniProt protein databases (E-value ≤10^−10^): 17β-hydroxysteriod dehydrogenase (*hsd17b3*), UDP glycosyltransferase 8 (*ugt8*), solute carrier family 6 (proline IMINO transporter) member 20 (*slc6a20*; also BLASTs to zebrafish *slc6a19b*), and a novel gene with unknown function. Larger numbers of sex-biased genes have been reported in the brains of other fishes (e.g., zebrafish [96], seabream [20], and black-faced blenny [159]). We conducted differential expression analysis at the isoform (represented by contigs) level with the most conservative software (DESeq) and stringent cut-offs (BH adjusted *p* value below 0.05) in order to reduce false positives [160]. We also included six intersex samples from the same experimental group in dispersion estimation and read count normalization (see “Methods” section). Such stringent analyses are likely to detect fewer but more reliable sex-biased contigs.

17β-hydroxysteroid dehydrogenase (Hsd17b3), the enzyme converting androstenedione to testosterone, was significantly up-regulated at the transcriptional level in the forebrain/midbrain of TP males (Fig. 8b and Additional file 3). Analyses in zebrafish have also shown male-biased expression of *hsd17b3* at the whole-brain level [96], suggesting conserved sex differences in local testosterone production in the brain. In teleosts, testosterone in the brain can be converted to E_2_ by the brain isoform of aromatase (*cyp19a1b*) or to 11-KT by Cyp11b and Hsd11b2 [161]. Although not significantly different after BH correction, *cyp19a1b* showed a 1.9-fold up-regulation in female brains, while *hsd11b2* was 1.7-fold higher in TP male brains (Fig. 8b and Additional file 3), suggesting a potentially higher E_2_ synthesis in female brains and 11-KT synthesis in TP male brains. Also, not significant after BH correction but likely biologically relevant, estrogen receptor 1 (*esr1*) and *esr2b* were up-regulated in TP male brains compared to female brains (Fig. 8b and Additional file 3). These patterns suggest that local neurosteroid production and signaling likely contribute to sex differences in the brain [161, 162] and are consistent with the previously documented influences of estrogen on behavioral sex change in bluehead wrasses [163].

The significance of the sexually dimorphic patterns of expression for other genes uncovered in the brain of the bluehead wrasse is unclear. *Ugt8* was significantly up-regulated in the forebrain/midbrain of TP males, while *slc6a20* showed an opposite pattern (Fig. 8b and Additional file 3). Wong et al. [96] also found *ugt8* to be up-regulated at the whole-brain level in male zebrafish. In mammals, UGT8 synthesizes galactocerebrosides, a major component of the myelin sheath surrounding nerves [164, 165]. Knocking out *ugt8* in mice reduces myelin thickness and nerve conduction, resulting in tremor and motor weakness [166, 167]. SLC6A20 transports proline and other imino acids and N-methylated amino acids across cell membranes [168]. Proline has been implicated in neuromodulation [169] and has been shown to modulate glutaminergic neurotransmission in mammals [170, 171]. There is currently no information on distribution or function of UGT8 and SLC6A20 in teleost brains. Sex differences in *ugt8* and *slc6a20* expression within the forebrain/midbrain of bluehead wrasses may translate into differences in neurotransmission and behavior, but these possibilities require more research to address.

We did not find significant differences in the expression of a number of key neuropeptide genes (Fig. 8b and Additional file 3), including arginine vasotocin (*avt*), isotocin (*it*), gonadotropin-releasing hormone (*gnrh*), and kisspeptin (*kiss*), that are known to be involved in socio-sexual behavior and/or reproduction in teleost fishes [172–175] and implicated in the regulation of socially induced sex change (reviewed in [55, 62]). *Avt* and *it* mRNAs are highly expressed in both TP male and female brains, but only *it* showed male-biased expression in our dataset, although this sex difference in *it* expression was not statistically significant after BH correction (Fig. 8b and Additional file 3). *Avt* mRNA expression was shown to be male-biased in the magnocellular preoptic area of bluehead wrasses [176] and to increase with behavioral sex change [64], but such differences may be masked due to the lower neuroanatomical resolution of whole-forebrain/midbrain sampling. The role of isotocin in sex change is less studied. However, it was shown that the number of isotocin-immunoreactive neurons in the preoptic area of bluebanded gobies (*Lythrypnus dalli*), a bi-directional sex-changing species, was higher in females than in males [177]. This sex-biased pattern appears to be opposite to our data for bluehead wrasses, although future studies are needed to determine if TP males have significantly higher expression than females and if isotocin plays a major role in regulating sex change.

## Conclusions

The genetic basis of sexual dimorphism in teleost fishes and the molecular mechanisms underlying the protogynous and protandrous sex change common to teleosts remain to be fully elucidated. In this study, we took advantage of high-throughput sequencing technology to generate the first high-quality transcriptome for a protogynous fish, the bluehead wrasse. This resource will make future comparative and experimental analyses of protogynous sex change possible. We also identified a large number of genes that exhibit sexually dimorphic expression in the gonad and several sex-biased genes in the forebrain/midbrain of bluehead wrasses. These genes include most known vertebrate sex-related genes as well as numerous novel genes that currently lack annotation but may well have important biological roles in sex differentiation and/or sex change. In addition, we find that most candidate genes implicated or known to be involved in sex determination and differentiation in other vertebrate systems showed conserved expression patterns in the bluehead wrasse with a few exceptions. This suggests that some subtle variability in the standard sex-determination regulatory network, although having evolved from a conserved toolkit, could be responsible for the sexual plasticity in these fishes. Overall, this study provides not only key data on the molecular basis of sexual dimorphism in the brain and gonad of bluehead wrasse, but also valuable resources for investigating the molecular pathways that underpin this extraordinary example of sexual plasticity in response to environmental influences. Further examination of the gene expression dynamics across the process of protogynous sex change will uncover the genetic cascade that progressively re-engineers a female into a male.

### Availability of supporting data

All sequencing data have been uploaded to NCBI Sequence Read Archive under accession number SRP06302.

## Additional files

Additional file 1: Table S1. Ensembl zebrafish gene IDs of sex-biased contigs in the gonad for GO and pathway enrichment analysis in DAVID. Additional file 2: Table S2. Results of differential expression analysis for the gonad (blue: male-biased contigs, red: female-biased contigs). Additional file 3: Table S3. Results of differential expression analysis for the brain (blue: male-biased contigs, red: female-biased contigs).
